# Immune Profiling in Early Cognitive Disorders (IMPRINT) study protocol: a longitudinal cohort study exploring biomarkers of inflammation in early dementia with Lewy bodies and Alzheimer’s disease, as part of the Dementias Platform UK

**DOI:** 10.1136/bmjopen-2025-107399

**Published:** 2025-11-21

**Authors:** Harry Crook, Peter Swann, Haddy Fye, Stacey Kigar, George Savulich, Anna Mckeever, Elena Herrero, Lordina Turner, Lina Aimola, Gabrielle D Grey, Dan Blackburn, Paul M Matthews, Li Su, Leonidas Chouliaras, James B Rowe, Paresh Malhotra, John T O’Brien

**Affiliations:** 1 Department of Clinical Neurosciences, University of Cambridge, Cambridge, UK; 2 UK Dementia Research Institute at Cambridge, Cambridge, UK; 3 Department of Psychiatry, University of Cambridge, Cambridge, England, UK; 4 Department of Medicine, University of Cambridge, Cambridge, England, UK; 5 Department of Brain Sciences, Imperial College London, London, UK; 6 The University of Sheffield, Sheffield, England, UK; 7 UK Dementia Research Institute at Imperial College London, London, UK; 8 Rosalind Franklin Institute, Didcot, UK; 9 Division of Neuroscience, The University of Sheffield, Sheffield, England, UK; 10 Medical Research Council Cognition and Brain Sciences Unit, Cambridge, UK

**Keywords:** Dementia, Immunophenotyping, Old age psychiatry

## Abstract

**Abstract:**

**Introduction:**

Growing evidence points towards the integral role of both central and peripheral inflammation across all neurodegenerative diseases, including dementia with Lewy bodies (DLB) and Alzheimer’s disease (AD). The immune alterations observed in these diseases may occur long before the onset of clinical and cognitive symptoms; however, the exact timing and role of inflammation in the pathogenesis of neurodegenerative disease remains unclear. Findings to date are conflicting, with most work focused on AD rather than other dementias and most studies from single sites and cross-sectional. Through longitudinally examining detailed phenotypes of the peripheral immune system using mass cytometry, the Immune Profiling in Early Cognitive Disorders study aims to uncover specific immune signatures in early AD and DLB, how these signatures change over time and how they relate to disease progression and cognitive changes.

**Methods and analysis:**

Blood, cerebrospinal fluid, saliva and urine samples will be collected from a cohort of participants with either prodromal (mild cognitive impairment) or early dementia due to Lewy bodies or AD (MCI-LB and DLB; and MCI-AD and AD), alongside healthy controls. Through immunophenotyping with mass cytometry, detailed immune fingerprints will be identified for these groups. We will assess which key combinations of immune cell clusters are predictive of disease phenotype, cognitive decline and progression to dementia. Samples will also be evaluated with novel techniques to measure markers of degenerative pathology and inflammation.

**Ethics and dissemination:**

This study was approved by the Preston North West Research Ethics committee (21/NW/0314) and is registered with the ISRCTN registry (ISRCTN62392656). The study is ongoing (since June 2022). Baseline visits are being undertaken, and follow-up visits have started for some participants. Full data analyses will be completed and submitted for publication upon conclusion of the study.

**STRENGTHS AND LIMITATIONS OF THIS STUDY**

- Longitudinal design allows the exploration of peripheral biomarkers of inflammation and the assessment of how these change over time and associate with disease progression.
- Cutting-edge technology: this study uses mass cytometry to profile immune cells. This technology allows for a precise quantification and detailed characterisation of cells.
- Measuring central and peripheral measures of inflammation simultaneously allows the exploration of the interplay between peripheral and central immune changes in neurodegeneration.
- This study will recruit participants based on clinical research criteria, meaning only those who required biomarker testing for clinical reasons will have these at the study outset; however, this will better reflect a more genuine memory clinic population.
- There is no neuroimaging component to this study; while this widens access and is keeping up with a shift to blood biomarkers, it does not lead to direct associations between inflammatory markers and neuroimaging findings.

## Introduction

### Background

 944 000 people in the UK are estimated to be living with dementia,^1^ which is the leading cause of death in the UK and many other countries.^2 3^ Alzheimer’s disease (AD) and Lewy body dementia (LBD) are two of the most common dementia-causing diseases, accounting for around 55% and 20% of dementia cases, respectively, in people over the age of 65.^4^ While specific neurodegenerative disorders present clinically with defined characteristic symptoms,^5 6^ recent research has revealed an increasing diversity of both distinct and overlapping pathological processes between different neurodegenerative diseases.^7 8^

The most frequent cause of dementia is AD, which is pathologically defined by the gradual deposition of interneural fibrillar amyloid-beta (Aβ) plaques and intraneural mis-folded τ protein tangles.^9^ Accumulation of these proteins within the brain parenchyma is followed by degeneration of areas of the temporal, parietal and occipital lobes, resulting in clinical presentation of a range of amnestic, visuospatial and language symptoms.^6 9^ The density and distribution of pathological Aβ and τ proteins can be measured in vivo using tools including positron emission tomography (PET) imaging and quantified by cerebrospinal fluid (CSF) sampling.^9^ Blood biomarkers to accurately measure these proteins in the context of AD are currently being developed.^10^

Dementia with Lewy bodies (DLB) is included within the umbrella of LBD, which comprises both DLB and Parkinson’s disease dementia (PDD). DLB and PDD overlap pathologically and are demarcated clinically by the 1 year rule of symptom onset, based on the temporal onset of cognitive and motor symptoms.^11^ A DLB diagnosis is given when cognitive symptoms precede motor symptoms by 1 year or more, while PDD is diagnosed when motor symptoms establish 1 year or more before cognitive ones. Pathologically, LBD is characterised by the presence of misfolded α-synuclein protein containing Lewy bodies within neurons, resulting in subcortical neurodegeneration including striatal dopaminergic neuronal loss^12^ but relative sparing of the medial temporal lobes.^5^ A particular cognitive profile is seen in DLB, typically including attentional and visuospatial impairment. The diagnostic criteria include the presence of cognitive fluctuations, visual hallucinations, parkinsonism and rapid eye movement sleep behaviour disorder, with supportive symptoms including autonomic dysfunction and depression.^5^ Both clinical AD and DLB are preceded by prodromal stages, termed mild cognitive impairment (MCI) due to AD/LB (MCI-AD/MCI-LB), with published standardised research criteria.^13 14^

 Although distinct diseases, AD and DLB share overlapping aspects of their aetiology, including vascular changes,^15 16^ mixed pathology^17^ and neuroinflammation.^18 19^ There are several different lines of evidence converging on neuroinflammation as a key pathophysiological process in several neurodegenerative diseases, including dementia. Epidemiological studies demonstrate a protective effect of long-term use of anti-inflammatories, such as non-steroidal anti-inflammatory drugs,^20^ while genome-wide association studies have identified polymorphisms in several inflammatory signalling pathways, including interleukin-1β, tumour-necrosis factor-α (TNF-α), triggering receptor expressed on myeloid cells 2 (TREM2) and chitinase-3-like protein 1 (YKL40),^18 19 21^ and other microglial risk genes.^22^,^25^ Pathologically, increased markers of microglial activation and transcriptional alterations are common features in neurodegenerative disease at postmortem.^26^,^28^ Neuroinflammation, as measured by PET imaging of the translocator protein (TSPO), has been found to be increased in study participants with MCI-AD^29^ and participants with mild DLB^30^ compared with controls; in AD, TSPO binding is associated with brain network dysfunction^31^ and future cognitive decline.^32^ A dynamic role of microglia and their activation across the AD trajectory has also been hypothesised.^33^ Furthermore, peripheral markers of inflammation point towards a role of inflammation in neurodegeneration. For example, high C-reactive protein levels in blood plasma have been associated with an increased risk of memory and visuospatial impairments,^34^ plasma TNF-α is negatively associated with brain volume^35^ and glial fibrillar acidic protein is elevated in MCI-AD and LBD.^36^ Together, these studies suggest that the immune system plays a key role in many neurodegenerative diseases, highlighting that neuroinflammation may be both an early disease marker and a therapeutic target for AD and DLB. Early therapeutic studies aimed at targeting the immune system show promising results in preclinical studies, and while initial early clinical trials have not shown efficacy,^37^,^45^ there are a wide range of more targeted immune modulatory drugs in clinical trials.^46^ Robust and clinically relevant immune biomarkers for early disease stages are urgently needed to ensure future success in these trials.

 Much of the previous work undertaken to identify peripheral biomarkers of inflammation in neurodegenerative diseases has focused on cytokines, whether in CSF or blood. While meta-analyses have highlighted significant differences in peripheral cytokine concentrations between people with dementia and controls,^47 48^ individual studies demonstrate inconsistent results which do not clearly link biomarkers to clinical phenotypes. For example, CSF concentrations of interleukin (IL)-6 were lower in participants with DLB compared with participants with AD and controls in one study,^49^ while other studies have found higher peripheral levels of IL-6 in DLB compared with controls,^50 51^ and another study has observed no differences in CSF IL-6 (or IL-1β) among DLB, AD or control groups.^52^ Further inconsistent findings, including no observable differences between disease-group participants and controls in peripheral IL-6 or other cytokines, have also been reported.^53^,^55^ Greater peripheral levels of IL-6 have been associated with worse cognitive performance in both DLB and AD,^48 49 56^ while TNF-α concentrations were related to severity of neuropsychiatric symptoms in DLB.^56^ Further challenges arise when comparing central and peripheral immune markers given the blood–brain barrier as an impediment to free exchange of cytokines, and the distinct nature of the neuroimmunological niche, with a lack of a relationship between central and peripheral inflammation seen previously.^30^

With distinct pathologies, it is possible that AD, DLB and other neurodegenerative diseases have specific peripheral immune profiles. Indeed, in CSF, IL-8 has been found to be higher in AD than in controls, with no difference seen between controls and DLB.^55^ Moreover, in AD, CSF concentration of YKL40 is increased^57^ but is not elevated in people with DLB or MCI-LB compared with controls,^58^ while soluble TREM2 and progranulin were not elevated in DLB unless AD pathology co-existed in these individuals.^58^ A confounder is the dynamic nature of the immune system along the disease trajectory. For example, King and colleagues demonstrated that participants with MCI-LB or MCI-AD had significantly higher plasma levels of IL-1β, IL-2, IL-4 and IL-10, as well as lower concentrations of TNF-α than the control, DLB and AD dementia groups.^59^ In addition, a longitudinal decline in IL-1β, IL-2, IL-4, IL-10 and INF-γ in the same cohort of participants was associated with worsening of cognitive performance.^60^ Although few longitudinal studies exist, these observed changes associated with disease progression in both pro- and anti-inflammatory markers over time complicate the ability to understand the underlying disease mechanisms and, hence, the utility of the cytokines as accurate biomarkers.

Flow cytometry-based immunophenotyping of cytokine-producing white blood cells in AD, DLB and their prodromal counterparts is another popular way of characterising differences in the immune system that are associated with disease metrics. Cross-sectional studies characterising patient-derived peripheral blood mononuclear cells (PBMCs) have yielded a much richer profile of immune system changes during diseased states. Most previous research has focused on flow cytometry-based characterisation in the context of AD only. For example, work in AD has shown CD8+T cells^61 62^ and CD4+CD28 memory T cells^63^ CD11b+ neutrophils^64^ are increased, while CD4+CD28+CD27+ naïve T cells are reduced^63^ in comparison to controls. However, Amin and colleagues found that people with DLB exhibit reduced proportions of CD4+helper T cells and CD19+HLA-DR+ activated B cells in comparison with people with AD, further highlighting variation between AD and DLB.^50^ Individuals with MCI also exhibit an increased concentration of CD4+T cells compared with control participants.^61^ Circulating myeloid dendritic cells are depleted in AD compared with control and MCI participants, though MCI subjects who later convert to AD exhibit a significant decline in myeloid dendritic cells in parallel with conversion to dementia.^65^ Furthermore, higher concentrations of CD56+ CD3+ natural killer T cells^66^ are associated with a greater disease severity and a poorer cognitive performance on testing, while activated CD8+T-cell levels correlate with parahippocampal microstructural tissue damage, measured with diffusion tensor imaging.^61^ Interestingly, greater CD4+regulatory T-cell (Treg)^67^ and overall CD3+T-cell^66^ numbers independently demonstrate a positive correlation with performance on the mini-mental state examination (MMSE), signifying better performance. The positive association between Treg levels and cognition is highlighted by the higher proportion of Tregs seen in people with MCI compared with those with AD,^67^ further highlighting fluctuations in the immune system across disease progression. Moreover, in Aβ-positive MCI participants, increased circulating levels of myeloid and dendritic cells were increased compared with Aβ-negative, cognitively normal participants, whereas CD3+T cells were increased in Aβ-positive participants regardless of cognitive status.^68^ Due to technical challenges, very few studies have explored neutrophils in AD/DLB, with a study in AD finding increased CD11b+ neutrophils,^64^ which are associated with disease severity. Although greater clarification is required, the evaluation of peripheral immune cells could help distinguish between neurodegenerative diseases, such as AD and DLB and their prodromal forms,^63 67 69 70^ which will increase diagnostic accuracy and prove useful for disease staging.^66 71 72^

The recent development of commercially available mass cytometry kits, which implement heavy metal antibody conjugations in lieu of the fluorescent molecules used for conventional flow cytometry, allows for a greater number of antibody probes that can be applied in a single, standardised assay.^73^ Mass cytometry can be performed on small volumes of blood compared with conventional flow cytometry (~250 uL vs 9 mL), with minimal cell handling. This reduces technical artefacts and allows the inclusion of neutrophils, in addition to PBMCs.^74^ This affords greater opportunity to deeply characterise the peripheral immune signatures of neurodegenerative disorders like AD and DLB. In a cross-sectional study that used mass cytometry, participants with AD had increased numbers of peripheral blood CD8+T-effector memory CD45RA+ (ERMA) cells, which negatively associated with cognitive performance.^75^ The understanding of the impact of inflammation, but also the timing of its impact in AD and DLB, remains inadequate. It is, therefore, imperative to improve this understanding to enable earlier diagnosis and inform future clinical trials. Through longitudinally examining detailed phenotypes of the peripheral immune system using mass cytometry, the Immune Profiling in Early Cognitive Disorders study aims to uncover specific immune signatures in early AD and DLB, how these signatures change over time and how they relate to disease progression and cognitive changes.

### Methods and analysis

#### Summary

**Figure 1:**
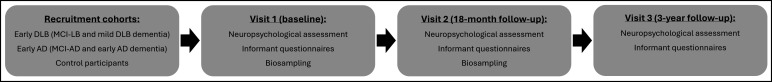
Flow chart detailing a participants’ journey through the study. *AD, Alzheimer’s disease; DLB, dementia with Lewy bodies; MCI-AD, mild cognitive impairment due to Alzheimer’s disease; MCI-LB, mild cognitive impairment due to Lewy bodies*.

 This protocol describes a longitudinal observational study consisting of a baseline, standardised, clinical and neuropsychological assessment followed by biomarker sampling including blood and optional CSF; saliva and urine will also be collected for future experimental assays. These baseline assessments will be repeated at the follow-up period of 18 months and longer-term (3 years) outcomes determined wherever possible (figure 1).

### Research questions

The main aim of this study is to perform deep immunoprofiling of immune changes in early AD and DLB compared with similarly aged healthy controls. The specific research questions are as follows:

1. Can differences in immunoprofiles in the blood and CSF be identified between participants with early AD and early DLB (which we define as including both prodromal and mild dementia) and similarly aged healthy control participants?
2. Are there longitudinal changes in these immune biomarkers over time, and how are these changes related to clinical features and disease progression?

### Patient and public involvement (PPI) statement

A patient and carer panel was formed during the design of the study, who provided feedback on the protocol and participant-facing materials that were incorporated into the study. This included reviewing the time and energy to complete questionnaires and the inclusion of lumbar punctures. During the study, all participants are sent a feedback questionnaire to provide an evaluation of their experience of the study and suggest recommendations for future research. The protocol has been presented at local PPI events in association with the NIHR Cambridge Biomedical Research Centre, and study participants are kept up to date with the study progress via a newsletter.

### Participants, recruitment and selection

 For early DLB and early AD groups, participants are to be recruited from cognitive disorder clinics in neurology, old age psychiatry and other related services at Cambridge University Hospital (CUH), Cambridge and Peterborough NHS Foundation Trust and Imperial College Healthcare NHS Trust. We will also seek referrals from other linked sites and recruit from the ‘Join dementia research’ (JDR) platform,^76^ where participants are able to travel to CUH or Imperial College Healthcare NHS Trust for biosampling. Control participants will be recruited from healthy older adults who express a willingness to participate in research via locally held research registers and JDR, as well as healthy friends and non-blood-related family members of patient participants who also indicate a willingness to participate.

 Potential participants will be provided with a copy of the participant information sheet and, following a period to consider these materials, will be contacted to ensure eligibility and suitability to partake in the study. An appointment will then be arranged for those willing and suitable to participate either for them to attend the study premises or to receive a home visit from a member of the research team to provide an opportunity to ask further questions and to obtain formal written consent from the participant. Consent for participation in the study and the sharing of pseudonymised data and samples will be taken. Due to the vulnerable nature of the study population and the longitudinal nature of the study, capacity to consent and participate will be considered throughout the study course. Clinicians and researchers recruiting to this study are trained in the assessment of capacity and capacity assessments will take place at each study visit. A potential consultee will be identified at the baseline research visit, and if a participant loses capacity during the study, the consultee process will be followed. Reimbursement for travel and meals will be provided for all study visits.

### Inclusion criteria

 Both male and female participants will be eligible for inclusion if they are over 50 years of age with a sufficient proficiency in English to allow for standardised cognitive testing. All participants except for healthy control subjects must have a reliable informant who is in sufficient contact to be able to complete questionnaires for informant-rated scales as well as provide a background history of the participating individual. DLB and AD group specific inclusion criteria for each cohort are outlined below under ‘Cohorts’. For the DLB cohort, a past diagnosis of Parkinson’s disease will not exclude participants, provided they meet the criteria for MCI-LB^14^ or DLB.^5^ Participants who meet the criteria for MCI, including those with MCI-LB and MCI-AD, will be recruited to the study. Wherever possible for the MCI group, we will seek to include people with biomarker evidence of LB or AD pathology. In the DLB and AD dementia groups, we will only include participants with mild dementia, as severely impaired participants are unlikely to comply with the study protocol. Mild dementia is defined in this study as a Clinical Dementia Rating Scale (CDR)^77^ of 0.5 or 1.

### Exclusion criteria

 Potential participants are excluded if they have concurrent major psychiatric illness, severe physical illness, neurological illness (apart from AD or DLB for patient-group participants) or any other co-morbidity that may limit their ability to fully participate in the study. Due to the fact that this study is exploring with particular interest the inflammatory cells and markers within the blood, any participant with inflammatory medical conditions, such as rheumatoid arthritis, or taking immunosuppressants, such as oral steroids, will be excluded from the study.

### Cohorts

 Participants will be recruited into three separate cohorts. The recruitment target detailed for each cohort allows for a 20% attrition rate over the course of the study. These cohorts will be:

1. Healthy control participants, defined as participants with MMSE scores of >26 and with the absence of any regular memory complaints/symptoms or any signs or symptoms suggestive of dementia.
2. Participants with early DLB include:
  1. Those who meet research criteria for the diagnosis of MCI-LB.^14^
  2. Those who meet criteria for mild DLB (CDR score of 0.5 or 1).^5^
3. Participants with early AD include:
  1. Those who meet diagnostic criteria for MCI-AD.^13^
  2. Those who meet diagnostic criteria for mild AD dementia (CDR score 0.5 or 1).^6^

### Overview of protocol

 Once written informed consent has been provided, participants will undertake an initial clinical assessment, including the collection of clinical and demographic information (current medication, smoking history, alcohol intake, education history, etc). Following this, a baseline visit consisting of a neuropsychological assessment battery, blood sampling (up to 80 mL) and an optional lumbar puncture to collect CSF (up to 20 mL) will be performed. Details of these procedures are outlined in depth below. Each participant will repeat these evaluations at 18 months and 3 years following the baseline visit.

### Clinical assessment, neuropsychological battery and informant questionnaires

 At all three study visits, detailed neuropsychological testing and informant questionnaires are undertaken using the battery outlined in table 1. The neuropsychological assessment battery is consistent between cohort groups in order to allow for between-group comparisons. The collection of clinical and demographical information is taken at the same time as the neuropsychological testing.

**Table 1:** Neuropsychological testing

Assessment type	Assessment name	Format	Purpose
Neuropsychological assessments	Addenbrooke’s cognitive examination revised^89^	Researcher-administered structured test	Multidomain cognitive screening tool
	Montreal Cognitive Assessment^90^	Researcher-administered structured test	Multidomain cognitive screening tool
	Trails A and B^91^	Researcher-administered structured test	Assessment of executive function
	Rey auditory verbal learning test^92 93^	Researcher-administered structured test	Assessment of verbal episodic memory
	Feeling of presence questionnaire^94^	Researcher-administered questionnaire	Assessment of presence hallucinations
	Noise Pareidolia Test^95^	Researcher-administered structured test	Assessment of visual hallucination-like illusions
Motor assessment	MDS-UPDRS part III (motor subscale)^96^	Performed by study clinician or trained researcher	Measure of Parkinsonism (motor aspects)
Psychiatric symptom questionnaires	Hospital Anxiety and Depression Scale^97^	A 14 item self-reported questionnaire	Assessment of symptoms of anxiety and depression
	Geriatric Depression Scale^98^	A 30 item self-reported questionnaire	Assessment of depressive symptoms
Informant questionnaires	Cambridge Behavioural Inventory, revised^99^	An 81-item carer-reported questionnaire	Assessment of several behavioural abnormalities in the everyday life including impulsivity and apathy
	Bristol Activities of Daily Living Score^100^	A 20-item carer-reported questionnaire	Measure of ability of person with dementia to carry out activities of daily living
	Neuropsychiatric Inventory^101^	Researcher administered, carer-reported 13 item screening tool	Assessment of psychopathology in people with brain disorders
	Clinician Assessment of Fluctuation^102^	Researcher administered, carer-reported with two screening questions and two rating questions	Assessment of conscious level and degree of symptomatic arousal fluctuation
	One Day Fluctuation Assessment Scale^102^	Researcher administered, carer-reported seven item tool	Assessment of conscious level and degree of symptomatic arousal fluctuation
	Dementia Cognitive Fluctuation Scale^103^	Researcher administered, carer-reported six item screening tool	Assessment of cognitive and functional symptomatic fluctuation
Other	Farnsworth Dichotomous Colour Vision Test^104 105^	Participant-led test	Assessment of dichotomous colour vision
	Brief Smell Identification Test^106 107^	12-item researcher-led test	Assessment of sense of smell and ability to recognise familiar odours

MDS-UPDRS, The International Parkinson and Movement Disorder Society sponsored revision of the Unified Parkinson’s Disease Rating Scale.

### Blood samples

 Up to 80 ml of blood will be drawn during the baseline and 18 month visits. These collections will occur as far as is practicable in the morning (between 9 am and 11 am) with the participant in a fasted state. Blood samples will be examined with a detailed immunophenotyping protocol which uses multi-parameter mass cytometry—a powerful technology for the analysis of population dynamics, cellular phenotype and function in the immune system at the single cell level.^73^ A comprehensive panel of antibodies tagged with heavy metal ions will be used to identify phenotypic and functional cell surface markers^73^ in whole blood on the day of sample collection. Samples prepared for mass cytometry will then be frozen and thawed prior to acquisition in batches on a mass cytometer, according to our previously published methods.^78^ This process systematically evaluates the unique repertoires of cell subsets and activation markers to provide a detailed map of an individual’s immune landscape. Following cleaning and quality control of the data, bioinformatic approaches that hierarchically cluster and quantify phenotypically similar cells will be applied for the analysis pipeline.^79^ In a subgroup of participants, fresh blood will be used for stimulation studies to investigate protein and metabolite production by cells belonging to different disease groups following immune challenge.^80^ Routine laboratory bloods will be undertaken to check clotting factors for safety and eligibility for CSF collection via an optional lumbar puncture. PBMCs will be collected and stored in liquid nitrogen for future validation assays. The remaining blood will be centrifuged to produce serum and plasma samples to be stored at −80°C for further testing of inflammatory and neurodegenerative markers in the future, while remaining cells will be stored for future genetic and transcriptional analyses.

### CSF samples

 The optional collection of CSF is expected to be consented to by approximately 20–25% of participants. Upon receiving informed consent, up to 20 ml of participant CSF will be collected through a lumbar puncture. If CSF collection is being obtained as part of the NHS diagnostics, and people are eligible for and consent to take part in this study, to avoid the need for a second lumbar puncture, up to 10 ml of CSF will be collected and stored for research purposes at the time of the NHS lumbar puncture. The lumbar puncture is optional, and participants will be provided with detailed written information on the procedure. All participants will discuss the procedure with a clinician who will perform an individual safety assessment. The safety of lumbar punctures in this population is well documented,^81^ with headache as the most common adverse event (occurring after 0.9–9% procedures, with more than 80% requiring no treatment). Serious adverse events such as infection or bleeding occurring in less than 1/10 000 procedures. The analysis protocols regarding immunophenotyping of blood samples mentioned above will be repeated with the CSF samples. Remaining CSF will also be analysed for biomarkers of infection, inflammation and neurodegeneration, which may provide information about the presence or absence of disease or relate to disease prognosis or severity. Collecting multiple samples from the same participants (ie, blood and CSF) will significantly enrich the study due to the multimodal data, allowing for a more complete profile of immune changes involved in people with cognitive impairment. For example, a key scientific question is the relationship between peripheral and central inflammation, and by collecting paired blood and CSF in a subset of participants, we will be able to contribute to this important area.

### Saliva samples

 In a subgroup of participants, saliva will be collected in a resting, unstimulated state by the participant drooling into a 50 mL collection vial with the head tilted forward, allowing the saliva to accumulate in the mouth. 5–20 mL will be collected within a 10-min time frame following a 1 hour period of not eating or drinking. Following processing, saliva samples will undergo a number of advanced analytical procedures to identify markers of inflammation, τ, phosphorylated-tau, Aβ and α-synuclein.

### Urine samples

 During study visits, 20–30 mL of mid-stream urine will be collected in sterile containers and will also be assessed for markers of inflammation, infection and degenerative changes. Following analysis protocols, remaining urine samples will be stored for future analysis.

### Analysis

 The authors have an extensive background of completing and analysing data from longitudinal research studies in these and similar populations. For example, the NIMROD study^82^ consisted of deep phenotyping and neuroimaging with longitudinal cognitive assessments, exploring the role of brain inflammation in dementia.^83 84^ The aim of this study is to detect differences in immune profiles between cohorts. The continuous variable of cognitive decline, as measured by the battery of neuropsychological assessments, as well as the binary progression to dementia from MCI, is also the outcome of interest. Multivariate statistical techniques alongside high-dimensional clustering, annotation and differential analysis^85^ will be employed to analyse immune cell counts and clinical measures in order to identify key combinations of immune cell clusters that are predictive of disease phenotype and rate of decline.^86^ This procedure will also reduce the dimensionality of the data. Alongside this, standard automated and/or manual gating, followed by parametric/non-parametric tests, will be applied, depending on the distribution of the data sets. Markers of inflammation, τ, phosphorylated-tau, Aβ and α-synuclein from bio-samples, will be compared using standard parametric methods. Cognitive and biomarker data will also be compared between healthy control, MCI-DLB/mild DLB and MCI-AD/mild AD groups in order to identify any group-level differences. Neuropsychological test scores will be converted to domain z scores for correlational analysis with biomarker and clinical data. Repeat measures from follow-up assessments will be normalised to control scores at baseline and change scores correlated with baseline changes to determine predictors of decline. If multiple predictors of decline are identified, regression and other multi-variate statistical methods including mixed effects models will be used to determine the key predictors. Genetic analysis will be used for confirmatory genetics of alleles known to be associated with inflammation or neurodegeneration and for exploratory analyses within collaborative studies.

### Power calculation

Our aim is for the study to have 80% power to detect differences in proportions of immune cell populations between groups at an alpha of 0.05. For an analysis of covariance with three groups and two covariates (age and sex), a sample size of 90 (30 in each group) would give sufficient power to detect an effect size of 0.33 (Cohen’s f, equivalent to Cohen’s d ~0.35). A sample size of 180 (60 in each group) would detect an effect size of 0.23 (Cohen’s f, equivalent to Cohen’s d ~0.24). By using data from previously published work performed by this group, which investigated myeloid cell subsets using flow cytometry in the NIMROD cohort,^87^ we estimated the effect sizes for significant immune cell differences between DLB, AD and controls to be between Cohen’s D 0.5–0.8. This suggests that sample sizes above 30 per group would be sufficient to detect these differences, with larger sample sizes detecting more subtle differences.

For correlations with cognition, Gate *et al*^75^ identified with mass cytometry immune cell populations in AD correlating with cognitive scores with slopes of magnitude ~0.5. A similar magnitude of correlations (r = 0.37–0.64) has been shown with immune cell subsets detected by mass cytometry and cognitive decline in AD.^88^ To detect correlations of this magnitude would require a sample size of 29 in each group (80% power at alpha = 0.05). A sample size of 60 in each group would detect the more modest correlations ~0.35 (all power calculations performed in G*Power v3.9.7.1).

### Ethics and dissemination

This study protocol has been approved by the Preston North West Research Ethics committee (reference 21/NW/0314). This study is registered with the ISRCTN registry (registration number ISRCTN62392656). The study is ongoing (since June 2022). Baseline visits are being undertaken and follow-up visits have started for some participants. Full data analyses will be completed upon conclusion of the study and submitted for presentation at international conferences and publication in peer-reviewed journals. Findings will also be shared with study participants and their relatives at local and regional patient and public involvement events in person and in regular newsletter updates. Written feedback will be sought from participants about their experience taking part in the study.
