# Supplementary material for: Immune Profiling in Early Cognitive Disorders (IMPRINT) study protocol: a longitudinal cohort study exploring biomarkers of inflammation in early dementia with Lewy bodies and Alzheimer’s disease, as part of the Dementias Platform UK
Source: BMJ Open. 2025 Nov 21;15(11):e107399. doi: 10.1136/bmjopen-2025-107399 (PMC12658555; doi:10.1136/bmjopen-2025-107399)
Supplement: Supplementary file 1 [file bmjopen-2025-107399.reviewer_comments.pdf]

PEER REVIEW HISTORY

BMJ Open publishes all reviews undertaken for accepted manuscripts. Reviewers are asked to complete a checklist review form and are provided with free text boxes to elaborate on their assessment. These free text comments are reproduced below.

ARTICLE DETAILS

Title (Provisional)

Immune Profiling in Early Cognitive Disorders (IMPRINT) study protocol: a longitudinal cohort study exploring biomarkers of inflammation in early dementia with Lewy bodies and Alzheimer's disease, as part of the Dementias Platform UK

Authors

Crook, Harry; Swann, Peter; Fye, Haddy; Kigar, Stacey; Savulich, George; Mckeever, Anna; Herrero, Elena; Turner, Lordina; Aimola, Lina; Grey, Gabrielle D; Blackburn, Dan; Matthews, Paul M; Li, Su; Chouliaras, Leonidas; Rowe, James B; Malhotra, Paresh; O'Brien, John

---

VERSION 1 - REVIEW

**Reviewer**	**1**
**Name**	**Bacsu, Juanita-Dawne**
**Affiliation**	**Thompson Rivers University, School of Nursing**
**Date**	**30-Sep-2025**
**COI**	

Thank you for the opportunity to review the protocol entitled, “Immune Profiling in Early Cognitive Disorders (IMPRINT) study protocol: a longitudinal cohort study exploring biomarkers of inflammation in early dementia with Lewy bodies and Alzheimer’s disease, as part of the Dementias Platform UK.” This dementia research protocol is timely and important because it aims to identify specific immune signatures in early Alzheimer’s disease and dementia with Lewy bodies, how these signatures change over time, and how they relate to disease progression and cognitive change. I have included my suggestions and edits below, and I hope that the authors find them helpful.

1. On page 2 of 21, please remove the period at the end of the article title, Immune Profiling in Early Cognitive Disorders (IMPRINT) study protocol: a longitudinal cohort study exploring biomarkers of inflammation in early dementia with Lewy bodies and Alzheimer’s disease, as part of the Dementias Platform UK.

2. In the abstract and throughout the paper, consider rephrasing the word “patients” to “people” or “participants.” A growing number of dementia advocates, journals, and funding

agencies are recognizing that terms such as “patients” can be viewed as stigmatizing as it implies dependency rather than a person-centered lens, especially since you are focusing on people living with early-stage dementia.

3. On pages 4-7 of 21, there is an excellent review of the background literature and justifying the rationale of the study, no edits required.

4. Study objective is clearly identified in the introduction section on page 7 of 21, lines 15-20 – “Through longitudinally examining detailed phenotypes of the peripheral immune system using mass cytometry, the Immune Profiling in Early Cognitive Disorders (IMPRINT) study aims to uncover specific immune signatures in early AD and DLB, how these signatures change over time, and how they relate to disease progression and cognitive changes.”

5. In the abstract if the word count allows, the objective would be strengthened by using similar wording to the introduction’s objective as it is easier to follow the longitudinal goals of the study.

6. On page 7 of 21, lines 23-34, the research questions would be better placed under the Methods and Analysis section, as they feel somewhat out of place in the introduction. Moving the questions would improve the flow of the paper.

7. On page 8 of 21, lines 24-27, it would be useful to expand on the informed consent process given that people with dementia are often viewed as a vulnerable population group. For example, how will capacity be assessed for participants with dementia? What measures will be in place if a participant’s capacity deteriorates over the 3 years in the longitudinal study, and will a continual consent process be used? Are there any honorariums/reimbursements provided to the person living with dementia and/or their care partners for their time, travel, and participation in the study?

8. The use of CSF lumbar puncture raises important ethical considerations due to its invasiveness, associated pain, and the significant risk of infection. Moreover, with multiple types of biological data being collected (e.g., blood, saliva, urine, and cerebrospinal fluid) it is important to provide more detail about how all of these samples will be analyzed and integrated into the study to justify their necessity.

9. It would also be helpful to know whether the authors have conducted similarly ambitious longitudinal studies in the past to address the feasibility of this proposed work.

10. In the Ethics and dissemination section on page 13 of 21, it would be helpful to know how the study’s findings will be shared with the participants living with dementia and their care partners. Currently, this discussion only mentions dissemination via academic conferences and journal articles but it would be helpful to know how the participants will be informed about the study findings.

VERSION 1 - AUTHOR RESPONSE

Response to reviewers

We thank the reviewer for their consideration of our protocol paper and their useful comments to improve it. Below we describe the steps we have taken to address each comment suggested by the reviewer in the format of "comment" followed by "response" for each. We have also added the text from the protocol paper as it has been updated, with changes highlighted in yellow.

Comment 1: On page 2 of 21, please remove the period at the end of the article title, Immune Profiling in Early Cognitive Disorders (IMPRINT) study protocol: a longitudinal cohort study exploring biomarkers of inflammation in early dementia with Lewy bodies and Alzheimer's disease, as part of the Dementias Platform UK.

Response: Thank you for spotting this typo. The period has now been removed.

Comment 2: In the abstract and throughout the paper, consider rephrasing the word "patients" to "people" or "participants." A growing number of dementia advocates, journals, and funding agencies are recognizing that terms such as "patients" can be viewed as stigmatizing as it implies dependency rather than a person-centered lens, especially since you are focusing on people living with early-stage dementia.

Response: We thank the reviewer for highlighting this important point and fully agree with how the term "patients" can be stigmatizing, and participants is a more suitable term for research studies. Throughout the paper, the term "patient(s)" has been changed to either "*people*" or "*participants*" and can be seen highlighted in yellow.

Comment 3: On pages 4-7 of 21, there is an excellent review of the background literature and justifying the rationale of the study, no edits required.

Comment 4: Study objective is clearly identified in the introduction section on page 7 of 21, lines 15-20 – "Through longitudinally examining detailed phenotypes of the peripheral immune system using mass cytometry, the Immune Profiling in Early Cognitive Disorders (IMPRINT) study aims to uncover specific immune signatures in early AD and DLB, how these signatures change over time, and how they relate to disease progression and cognitive changes."

Response: We thank the reviewer for these comments and provide no further edits to these sections of the text.

Comment 5: In the abstract if the word count allows, the objective would be strengthened by using similar wording to the introduction's objective as it is easier to follow the longitudinal goals of the study.

Response: To strengthen the objective outlined in the abstract and clarify the longitudinal goals of the study, we have adopted similar wording to that used in the introduction, as suggested by the reviewer. The abstract now reads as:

"Introduction: Growing evidence points towards the integral role of both central and peripheral inflammation across all neurodegenerative diseases, including dementia with Lewy bodies (DLB) and Alzheimer's disease (AD). The immune alterations observed in these diseases may occur long before the onset of clinical and cognitive symptoms, however, the exact timing and role of inflammation in the pathogenesis of neurodegenerative disease remains unclear. Findings to date are conflicting, with most work focussed on AD rather than other dementias and most studies from single sites and cross-sectional. Through longitudinally examining detailed phenotypes of the peripheral immune system using mass cytometry, the Immune Profiling in Early Cognitive Disorders (IMPRINT) study aims to uncover specific immune signatures in early AD and DLB, how these signatures change over time, and how they relate to disease progression and cognitive changes.

Methods and analysis: Blood, cerebrospinal fluid, saliva, and urine samples will be collected from a cohort of participants with either prodromal (mild cognitive impairment) or early dementia due to Lewy bodies or AD (MCI-LB and DLB; and MCI-AD and AD), alongside healthy controls. Through immunophenotyping with mass cytometry, detailed immune fingerprints will be identified for these groups. We will assess which key combinations of immune cell clusters are predictive of disease phenotype, cognitive decline, and progression to dementia. Samples will also be evaluated with novel techniques to measure markers of degenerative pathology and inflammation.

Ethics and dissemination: This study was approved by the Preston North West Research Ethics committee (21/NW/0314) and is registered with the ISCTRN registry (ISRCTN62392656). The study is ongoing (since June 2022). Baseline visits are being undertaken and follow-up visits have started for some participants. Full data analyses will be completed and submitted for publication upon conclusion of the study."

Comment 6: On page 7 of 21, lines 23-34, the research questions would be better placed under the Methods and Analysis section, as they feel somewhat out of place in the introduction. Moving the questions would improve the flow of the paper.

Response: Thank you for this comment, we agree that the research questions are better placed under the "Methods and analysis" section, and we have now placed them here following the summary paragraph.

Comment 7: On page 8 of 21, lines 24-27, it would be useful to expand on the informed consent process given that people with dementia are often viewed as a vulnerable population group. For example, how will capacity be assessed for participants with dementia? What measures will be in place if a participant's capacity deteriorates over the 3 years in the longitudinal study, and will a continual consent process be used? Are there any honorariums/reimbursements provided to the person living with dementia and/or their care partners for their time, travel, and participation in the study?

Response: We have taken into consideration these suggestions and expanded the section discussing capacity and reimbursements to address the reviewer's concerns. The section now reads as follows:

"Consent for participation in the study and the sharing of pseudonymised data and samples will be taken. Due to the vulnerable nature of the study population and the longitudinal nature of the study, capacity to consent and participate will be considered throughout the study course. Clinicians and researchers recruiting to this study are trained in the assessment of capacity and capacity assessments will take place at each study visit. A potential consultee will be identified at the baseline research visit, and if a participant loses capacity during the study the consultee process will be followed. Reimbursement for travel and meals will be provided for all study visits."

Comment 8: The use of CSF lumbar puncture raises important ethical considerations due to its invasiveness, associated pain, and the significant risk of infection. Moreover, with multiple types of biological data being collected (e.g., blood, saliva, urine, and cerebrospinal fluid) it is important to provide more detail about how all of these samples will be analyzed and integrated into the study to justify their necessity.

Response: We agree with the reviewer that invasive procedures must be carefully considered based on the risks and potential scientific benefit, and in this study participant wellbeing, safety, and choice is a priority. We have added further details about the lumbar process consent in keeping with this:

"The optional collection of CSF is expected to be consented to by approximately 20-25% of participants. Upon receiving informed consent, up to 20ml of participant CSF will be collected through a lumbar puncture. If CSF collection is being obtained as part of the NHS diagnostics, and people are eligible for and consent to take part in this study, to avoid the need for a second lumbar puncture, up to 10ml of CSF will be collected and stored for research purposes at the time of the NHS lumbar puncture. The lumbar puncture is optional, and participants will be provided with detailed written information on the procedure. All participants will discuss the procedure with a clinician who will perform an individual safety assessment. The safety of lumbar punctures in this population is well documented (100) with headache the most common adverse event (occurring after 0.9-9% procedures, with more than 80% requiring no treatment). Serious adverse events such as infection or bleeding occurring in less than 1/10,000 procedures. The analysis protocols regarding immunophenotyping of blood samples mentioned above will be repeated with the CSF samples. Remaining CSF will also be analysed for biomarkers of infection, inflammation, and neurodegeneration, which may provide information about the presence or absence of disease or relate to disease prognosis or severity. Collecting multiple samples from the same participants (i.e. blood and CSF) will significantly enrich the study due to the multimodal data allowing for a more complete profile of immune changes involved in people cognitive impairment. For example, a key scientific question is the relationship between peripheral and central inflammation, and by collecting paired blood and CSF in a subset of participants we will be able to contribute to this important area."

Comment 9: It would also be helpful to know whether the authors have conducted similarly ambitious longitudinal studies in the past to address the feasibility of this proposed work.

Response: We thank the reviewer for this comment and we have now added some text demonstrating a similar longitudinal study undertaken previously. This section reads as:

"The authors have an extensive background of completing and analysing data from longitudinal research studies in these and similar populations. For example, the NIMROD study (101) consisted of deep phenotyping and neuroimaging with longitudinal cognitive assessments, exploring of the role of brain inflammation in dementia (102, 103)."

Comment 10: In the Ethics and dissemination section on page 13 of 21, it would be helpful to know how the study's findings will be shared with the participants living with dementia and their care partners. Currently, this discussion only mentions dissemination via academic conferences and journal articles but it would be helpful to know how the participants will be informed about the study findings.

Response: Thank you for this important comment. To address this, we have included a statement discussing how study finding will be shared with the participants and relatives:

"Findings will also be shared with study participants and their relatives at local and regional patient and public involvement events in person, and in regular newsletter updates. Written feedback will be sought from participants about their experience taking part in the study."
